# Golgi Anti-apoptotic Proteins Are Highly Conserved Ion Channels That Affect Apoptosis and Cell Migration*

**DOI:** 10.1074/jbc.M115.637306

**Published:** 2015-02-24

**Authors:** Guia Carrara, Nuno Saraiva, Maddy Parsons, Bernadette Byrne, David L. Prole, Colin W. Taylor, Geoffrey L. Smith

**Affiliations:** From the ‡ Department of Pathology, University of Cambridge, Cambridge CB2 1QP, United Kingdom,; the ‖ Department of Pharmacology, University of Cambridge, Cambridge CB2 1PD, United Kingdom,; the ¶ Division of Molecular Biosciences, Imperial College London, London SW7 2AZ, United Kingdom, and; the § Randall Division of Cell and Molecular Biophysics, King's College London, London SE1 1UL, United Kingdom

**Keywords:** Electrophysiology, Ion Channel, Lipid Bilayer, Membrane Protein, Viral Protein

## Abstract

Golgi anti-apoptotic proteins (GAAPs) are multitransmembrane proteins that are expressed in the Golgi apparatus and are able to homo-oligomerize. They are highly conserved throughout eukaryotes and are present in some prokaryotes and orthopoxviruses. Within eukaryotes, GAAPs regulate the Ca^2+^ content of intracellular stores, inhibit apoptosis, and promote cell adhesion and migration. Data presented here demonstrate that purified viral GAAPs (vGAAPs) and human Bax inhibitor 1 form ion channels and that vGAAP from camelpox virus is selective for cations. Mutagenesis of vGAAP, including some residues conserved in the recently solved structure of a related bacterial protein, BsYetJ, altered the conductance (E207Q and D219N) and ion selectivity (E207Q) of the channel. Mutation of residue Glu-207 or -178 reduced the effects of GAAP on cell migration and adhesion without affecting protection from apoptosis. In contrast, mutation of Asp-219 abrogated the anti-apoptotic activity of GAAP but not its effects on cell migration and adhesion. These results demonstrate that GAAPs are ion channels and define residues that contribute to the ion-conducting pore and affect apoptosis, cell adhesion, and migration independently.

## Teaser

**Background:** GAAPs regulate intracellular Ca^2+^ fluxes, cell migration, and apoptosis.

**Results:** GAAP forms a cation-selective channel, and residues involved in its ion-conducting properties were identified.

**Conclusion:** Mutations within the pore demonstrate that GAAP effects on apoptosis and migration are separable.

**Significance:** Characterization of the pore region of GAAP provides insight into the mechanism of action of this novel and highly conserved ion channel.

## Introduction

Golgi anti-apoptotic protein (GAAP),^4^ also known as TMBIM4 (transmembrane Bax (Bcl-2-associated X protein) inhibitor-1 motif-containing 4), is a highly conserved, hydrophobic protein present throughout eukaryotes. GAAPs from vertebrates, insects, and plants share a remarkable conservation of size, primary sequence, and hydrophobicity profile, suggesting highly conserved structures (1). Phylogenetic analyses suggest that GAAPs arose very early in eukaryotic evolution and that most members of the TMBIM family arose from a GAAP-like progenitor about 2,000 million years ago (2, 3). The human GAAP (hGAAP) was proposed to be an essential housekeeping protein based on microarray analysis (4), its ubiquitous expression in all human tissues tested, and its requirement for cell viability (1).

The GAAP gene, *6L*, was discovered in camelpox virus (CMLV) and encodes a protein with 237 amino acid residues (5) that is also present in cowpox virus (CPXV) and in 3 of 16 strains of vaccinia virus (VACV). In these viruses, the protein is very highly conserved (>98% amino acid identity). Although not essential for the replication of VACV in cell culture, vGAAP affected the virulence of the virus *in vivo*, indicating an important function (1). vGAAP and hGAAP share 73% amino acid identity, the same length, and a conserved hydrophobicity profile (1). This high degree of conservation between a VACV protein and a human orthologue is unusual, because such sequence identities are commonly only about 20–30% (6–8). hGAAP and vGAAP are so similar that expression of vGAAP can prevent apoptosis following knockdown of endogenous hGAAP with siRNA (1). Both vGAAP and hGAAP localize predominantly to the Golgi apparatus (1). hGAAP, vGAAP, and human Bax inhibitor-1 (hBI-1), another widely expressed TMBIM protein, share similar functions and secondary structures. The three proteins inhibit apoptosis induced by a wide range of stimuli (1, 9), and all contain a conserved UPF0005 motif (2, 10), both characteristic features of the TMBIM family.

The mRNA for hGAAP is significantly up-regulated in some human malignant tissues compared with normal tissues (11, 12). The high expression of hGAAP mRNA in glioblastoma multiforme tumors is associated with poor outcomes (12). Dysregulation of hGAAP in non-small cell lung carcinoma samples led to hGAAP being proposed as a novel candidate prognostic marker for this disease in patients who have never smoked (11). Expression of BI-1 is also dysregulated in some human malignant tissues, such as breast and prostate cancers (13–16), and overexpression of BI-1 increases cancer progression and metastasis in mice (17, 18). The structural and functional relatedness of hGAAP and BI-1 led us to suggest that hGAAP, like BI-1 (19), may be important for cancer development and a target for new anti-cancer drugs.

Overexpression of hGAAP lowers the Ca^2+^ content of the endoplasmic reticulum (ER) and Golgi apparatus, whereas inhibiting expression of endogenous hGAAP has the opposite effects (20). hGAAP also regulates focal adhesion dynamics and cell adhesion and migration and promotes Ca^2+^ influx across the plasma membrane via store-operated Ca^2+^ entry (21). However, the molecular mechanisms by which hGAAP affects Ca^2+^ fluxes are not known.

hGAAP does not interact with the sarcoplasmic/endoplasmic reticulum Ca^2+^-ATPase (SERCA) 2b that mediates Ca^2+^ uptake into the ER but was reported to interact with inositol 1,4,5-trisphosphate receptors (IP_3_Rs), through which it might affect Ca^2+^ homeostasis (20). Alternatively, GAAP may form an ion channel or exchanger directly. Overexpression of BI-1 reduces the Ca^2+^ content of intracellular stores (22) and mediates Ca^2+^/H^+^ fluxes when reconstituted into liposomes (22, 23). In addition, ionic currents have been recorded from the plasma membrane of cells overexpressing BI-1 (24).

Bioinformatic analyses of the membrane topology of hGAAP, vGAAP, and other TMBIM family members predicted that each protein has either six or seven transmembrane domains (TMDs) (25). However, topology maps of vGAAP, hGAAP, and hBI-1 deduced using antibody accessibility indicated that for all three proteins, the N and C termini were cytosolic, consistent with six TMDs linked by short interconnecting regions (25, 26). An epitope tag inserted within the hydrophilic region between the sixth and seventh hydrophobic regions was accessible to antibody, suggesting that the seventh hydrophobic region might form a re-entrant membrane loop near the charged C terminus (25). For all three proteins, pH affected their homo-oligomerization (22, 27).

While the present work was in progress, a high resolution structure of BsYetJ, a bacterial protein related to hBI-1 and hGAAP (18 and 21% amino acid identity, respectively), was published (28). This structure revealed seven TMDs, with TMD7 at the core of the structure. Increasing pH (from 6 to 8) caused a substantial lateral movement of TMD2, suggestive of channel gating (28). The first six of the seven α-helices in BsYetJ match their locations within the topological map of GAAPs (25, 28). However, the origin of the difference in the apparent organization of TMD7 between vGAAP, hGAAP, and BsYetJ is not clear. It is possible that attaching a hydrophilic tag, such as yellow fluorescent protein (YFP) or a hemagglutinin (HA) epitope, at the C terminus of GAAPs induced an aberrant topology, although GAAP tagged in this way retained its function as a regulator of apoptosis, adhesion, and migration (1, 21, 25). It is also possible that the crystallization conditions used for such a highly hydrophobic protein as BsYetJ might have altered its topology. Alternatively, there may be genuine differences in the membrane topologies of GAAPs/hBI-1 and the distantly related bacterial protein.

Here electrophysiological recordings from purified vGAAPs and hBI-1 reconstituted into lipid bilayers demonstrate that these proteins form ion channels and that vGAAP is selective for cations. Mutation of conserved residues toward the C terminus of vGAAP increased the single-channel conductance (E207Q or D219N) and changed the ion selectivity (E207Q). This suggests that these residues are located within the channel pore. Of these mutations, only E207Q reduced the effects of GAAP on cell migration and adhesion. Conversely, mutation of Asp-219, but not of Glu-207, abolished the anti-apoptotic effect of vGAAP. The ancient origins of GAAPs and their striking level of conservation suggest that these findings are likely to be relevant to other GAAPs and members of the TMBIM family.

## EXPERIMENTAL PROCEDURES

### 

#### 

##### Phylogenetic and Bioinformatics Analyses

Searches for GAAP orthologues or known ion channels with regions of sequence similarity to GAAP were carried out with BLASTP (29) using full-length or partial GAAP query sequences. Multiple amino acid sequence alignments were generated using ClustalW (30). For the generation of sequence conservation plots, ClustalW protein alignments were quantified using Scorecons (31) to generate a residue conservation score list. Hydrophobicity profiles were generated using the Kyte and Doolittle algorithm (32), and multiple profiles were overlapped using GraphPad Prism version 5.

The GenBank^TM^ accession numbers for known and putative GAAP orthologues discussed in this paper are as follows: CMLV (AAG37461.1), VACV strain Evans (AAV98625), CPXV (ADZ30397.1), *Homo sapiens* (AAF14868), *Canis lupus familiaris* (XP_531662), *Bos taurus* (AAI51433), *Rattus norvegicus* (AAH60596), *Gallus gallus* (XP_001235093), *Danio rerio* (NP_998303), *Trichoplax adhaerens* (XP_002108506), *Caenorhabditis elegans* (NP_509543), *Schizosaccharomyces pombe* (NP_588431), *Arabidopsis thaliana* (NP_193209), *Penicillium chrysogenum* (XP_002568682.1), *Campylobacter jejuni* (WP_002862428.1), *Helicobacter pylori* (WP_001240234.1), *Candidatus Chloracidobacterium thermophilum* (ABV27378.1), *Tuber melanosporum* (XP_002836789), *Cerapachys biroi* (EZA59874), *Tribolium castaneum* (XP_969476), *Genlisea aurea* (EPS68151), *Zea mays* (EGU84019), and *Saccharomyces cerevisiae* (NP_014094). The accession numbers of other proteins used are as follows: *H. sapiens* TMBIM1/Recs1 (AAH26693), *H. sapiens* TMBIM2/FAIM2 (Q9BWQ8), *H. sapiens* TMBIM3/GRINA (NP_001009184), *H. sapiens* TMBIM5/Ghitm (Q9H3K2), *H. sapiens* TMBIM6/BI-1 (P55061), *Bacillus subtilis* YetJ (O31539), *H. sapiens* RyR1 (P21817), *H. sapiens* Ca_v_2.1 (O00555), *Streptomyces lividans* KcsA (P0A334), *Bacillus cereus* NaK (2AHZ_A), and *Mus musculus* K_Ca_1.1 (Q08460).

##### Cell Culture and Transfection

U2-OS and HEK 293T cells were grown in DMEM (Life Technologies) supplemented with 10% fetal bovine serum (FBS), 50 units/ml penicillin, 50 μg/ml streptomycin, and 2 mm l-glutamine. Plasmid transfections used FuGENE 6 (Roche Applied Science) according to the manufacturer's instructions.

##### Expression Plasmids and Stable Cell Lines

Full-length CMLV GAAP was amplified by PCR with a 3′ HA epitope incorporated within the reverse primer and cloned into vector pcDNA3.1+ (Invitrogen) using restriction sites BamHI/EcoRI. Single amino acid mutations G152A, E178Q, E207Q, and D219N were inserted into CMLV GAAP-HA using the QuikChange multiple-site-directed mutagenesis kit (Stratagene) according to the manufacturer's instructions. PCR-based site-directed mutagenesis was performed using a forward primer containing each point mutation flanked with overhangs complementary to adjacent GAAP sequences. U2-OS cells were transfected with the empty pcDNA3.1+ vector or the vector encoding HA-tagged CMLV GAAP or its mutant forms. Transfected cells were selected for their resistance to 500 μg/ml neomycin (Invitrogen). The CMLV GAAP alleles were subcloned into a lentiviral bicistronic expression vector using restriction sites BamHI/EcoRI. HEK 293T cells were co-transfected with 0.5 μg of lentivirus packaging vector, 0.5 μg of vesicular stomatitis virus glycoprotein G-expressing vector, and 0.76 μg of lentiviral bicistronic vector coding for the gene of interest and GFP in the first and second cistron, respectively. After 72 h, virions produced in the supernatant were harvested, and cell debris was removed by centrifugation (300 × *g* for 5 min) and filtration (0.45-μm pore size filter). U2-OS cells at 50% confluence were infected with the lentivirus preparation, and GFP-expressing cells were sorted using a MoFloMLS high speed cell sorter (Beckman Coulter). The U2-OS cell line expressing FLAG-tagged Bcl-X_L_ was a gift from Dr. D. L. Veyer (University of Cambridge).

##### Immunoblotting

Cells were lysed at 4 °C in CHAPS lysis buffer (50 mm Tris-HCl, pH 7.5, 100 mm NaCl, 2 mm EDTA, 1% (w/v) CHAPS (Sigma-Aldrich), and protease and phosphatase inhibitor mixtures (Roche Applied Science)). The lysates were cleared by centrifugation (15,000 × *g* for 15 min), resolved on a 12% SDS-polyacrylamide gel, and transferred onto a nitrocellulose membrane. The antibody dilutions used were rabbit anti-FLAG (1:1,000; Sigma, F7425), rabbit anti-HA (1:10,000; Sigma, H6908), mouse anti-tubulin (1:10,000; Millipore, 05-829), rabbit anti-YFP/GFP (1:25,000; Abcam, ab290), and anti-SERCA (1:1,000; Calbiochem, 564702). Purified proteins were resolved on Novex 12% Tris/glycine gels (Invitrogen) in the absence of reducing agent and stained with Imperial stain (Pierce) or Coomassie Blue (R-250).

##### Immunoprecipitation

COS-7 cells at 70–80% confluence were transfected using Lipofectamine 2000 with empty pCI vector, plasmids encoding full-length or truncated IP_3_R1 with an N-terminal YFP tag (33), or YFP/GFP-tagged controls that localize to different organelles: pEYFP-ER (ER-YFP) (lumen of the ER; Clontech), YFP-lamin B1 (LamB1) (nucleus; Clontech), pEGFP-tubulin (GFP-Tub) (microtubules; Clontech), and pEYFP-C1 (cytosolic YFP; Clontech). 24 h after transfection, cells were infected at an multiplicity of infection of 3 for 16 h with recombinant VACV strain Evans lacking vGAAP (v-ΔGAAP) or with a C-terminally HA-tagged vGAAP (vGAAP Rev-HA) (1). Cells were harvested and lysed in 1% CHAPS buffer (50 mm Tris-HCl, pH 7.5, 500 mm NaCl, 2 mm EDTA, 1% CHAPS (w/v), protease inhibitors, and phosphatase inhibitors). After centrifugation (15,000 × *g*, 15 min, 4 °C), the supernatant was retained, and the total protein content was determined by a Bradford assay (Bio-Rad). Some of the supernatant was kept for gel analysis. After a 2-h incubation with protein G-Sepharose beads (Roche Applied Science), anti-GFP/YFP (1:200; Abcam) was added to the cell lysates and incubated overnight with gentle rocking. Protein G-Sepharose beads (40 μl) were then added, incubated for 2 h, and then washed four times with 1% CHAPS buffer. The whole cell extracts and immunoprecipitated proteins were resolved by SDS-PAGE and immunoblotted with anti-YFP/GFP, anti-HA, or anti-SERCA antibodies.

##### Apoptosis Assays

U2-OS cells were seeded in 96-well dishes at 10^4^ cells/well in DMEM supplemented with 10% FBS. After 48 h, cells were mock-treated or treated with staurosporine (STS) (0.5 μm, 6 h), doxorubicin (DOXO) (3 μm, 48 h), or cycloheximide (CHX) (20 μg/ml) and human tumor necrosis factor α (TNF-α) (10 ng/ml) for 16 h in DMEM with 2% FBS. Media used for mock treatments were supplemented with concentrations of the solvents used to dissolve drugs: DMSO for STS or DOXO and Milli-Q water for CHX and TNF-α. Caspase-3 and -7 activity was determined according to the manufacturer's instructions, using the Caspase-Glo 3/7 substrate (Promega). The resulting luminescence, which is proportional to caspase-3/7 activity, was measured using a luminometer (Omega).

##### Cell Spreading Assay

U2-OS cells were detached with trypsin treatment, rinsed twice in DMEM containing 1% FBS, and seeded onto coverslips coated with 10 μg/ml fibronectin (Invitrogen). After 30 min at 37 °C, cells were fixed with 4% paraformaldehyde in 250 mm HEPES, pH 7.4, for 20 min. Image acquisition was performed by confocal microscopy (LSM 510 META, Carl Zeiss) with a ×63, 1.4 numerical aperture oil immersion objective. Mean cell areas were determined using ImageJ (National Institutes of Health).

##### Random Cell Migration Assay

U2-OS cells were seeded at low density (30%) in DMEM containing 10% FBS on 10 μg/ml fibronectin-coated (Invitrogen) 12-well plates. Cells were allowed to adhere for 4 h at 37 °C. Image acquisition was performed within an environmental chamber at 37 °C at 5-min intervals for 8 h from six different fields in each well using a wide field microscope (LSM 5 PASCAL, Zeiss), a ×10 objective, and a camera (AxioCam HRm; Carl Zeiss). Migration tracks were generated using the ImageJ Manual Tracking plugin, and tracks were analyzed using a Mathematica 7 notebook written in-house (provided by G. Dunn, King's College London) to calculate migration rates.

##### Expression and Purification of Proteins

GAAPs and hBI-1 were expressed in *S. cerevisiae* strain FGY217 (34). The engineered proteins had a cleavable C-terminal GFP-His_8_ tag and were expressed from the p424GAL1-TEVp-GFP-His_8_ vector under control of the galactose promoter (35). The proteins were purified and analyzed according to a protocol developed for other transmembrane proteins (36) in 150 mm NaCl, 20 mm Tris-base, 5% glycerol, and 0.06% lauryldimethylamine *N*-oxide, pH 7.5. The GFP-His_8_ tag was cleaved by adding His_8_-tagged tobacco etch virus protease to the purified GFP-His_8_-tagged protein at a molar ratio of 1:1 and digested overnight at 4 °C. Cleaved GFP-His_8_ and the His-tagged protease were removed using a HisTrap nickel column (GE Healthcare), and the untagged target proteins were harvested from the flow-through. The purified proteins were concentrated using an Amicon Ultra centrifugal filter with a molecular mass cut-off of 30 kDa (Millipore) and analyzed on a Superdex 200 size exclusion chromatography (SEC) column (GE Healthcare). Fractions corresponding to the purified target protein were collected and concentrated to 1.5–2 mg/ml. Purified adenosine A_2A_ receptors (A_2A_Rs) were provided by Dr. S. Singh (37).

##### Reconstitution of Proteins into Giant Unilamellar Vesicles (GUVs)

GUVs were produced by electroformation from a mixture of 1:10 cholesterol (Sigma) to 1,2-diphytanoyl-*sn*-glycero-3-phosphocholine (Avanti Polar Lipids) dissolved in chloroform (Carl Roth). The lipid mixture (20 μl) was spread on the conductive side of an indium tin oxide-coated slide and allowed to dry for 10 min. The dried lipid film was covered with 1 m sorbitol (250 μl) enclosed within a greased O-ring and overlaid with a second indium tin oxide-coated slide with its conductive side facing the lipids. The assembly was then connected to a Vesicle Prep Pro (Nanion Technologies, Munich, Germany). The electric field parameters used for electroformation of GUVs were as follows: 5 Hz, 3 V, for 128 min at 20 °C. GUVs were then resuspended from the slide within the 1 m sorbitol overlay, collected, and stored at 4 °C for 3–4 days. Proteins were incorporated into GUVs by mixing purified protein (10 μl) with the GUV preparation (90 μl) (0.2 mg/ml final protein concentration). Bio-Beads SM-2 absorbents (152-8920, Bio-Rad) washed previously in methanol (3 × 10 min), ethanol (3 × 10 min), and Milli-Q water (6 × 5 min) were added (40 mg/ml, 15 min) and then removed three times during the reconstitution procedure (45 min total incubation) to remove excess detergent micelles. Protein-containing GUVs were stored at 4 °C and used within a few h for bilayer recordings.

##### Electrophysiological Recording

Single-channel recordings were performed with a Port-a-Patch system (Nanion Technologies) (38, 39) using NPC-1 borosilicate glass chips (5–10 megaohm resistance). Patch medium (5 μl) (140 mm KCl, 200 nm free Ca^2+^ (220 μm CaCl_2_ buffered with 0.5 mm BAPTA-Na_4_) and 10 mm HEPES-free acid, adjusted to pH 7 with KOH) was added to the *cis* side of the chip. Planar lipid bilayers were formed across the micrometer-sized aperture of the chip by suction after the addition of GUVs in 1 m sorbitol (5 μl) to the *cis* side (see Fig. 2*C*). The resistances were 1–10 gigaohms after formation of bilayers. Protein-reconstituted GUVs (5 μl) (in 15 mm NaCl, 2 mm Tris-base, 0.5% glycerol, 0.006% lauryldimethylamine *N*-oxide, and 0.9 m sorbitol, pH 7.25) were then added to allow incorporation of the purified protein into the bilayer. The final composition of the medium in the *trans* chamber (5 μl) was 140 mm KCl, 200 nm free Ca^2+^ (220 μm CaCl_2_ buffered with 0.5 mm BAPTA-Na_4_), 10 mm HEPES-free acid, adjusted to pH 7 with KOH. In the *cis* chamber (15 μl, ground), the final composition of the medium was 46.7 mm KCl, 200 nm free Ca^2+^ (73 μm CaCl_2_ buffered with 0.17 mm BAPTA-Na_4_), 5 mm NaCl, 3.33 mm HEPES-free acid, 0.67 m sorbitol, 0.67 mm Tris-base, pH 7 (see Fig. 2*C*). Recordings were acquired with PatchMaster software (Nanion Technologies) in the “on cell” mode, using an EPC 10 patch clamp amplifier (HEKA). Voltages are expressed as the potential on the *cis* side relative to the *trans* side. Single-channel currents are shown such that downward deflections (negative currents in the current-voltage curves) represent positive ions flowing from the *trans* to the *cis* side of the bilayer. For continuous current recordings, holding potentials were applied for 1-min intervals in increments of 20 mV or until bursts of spontaneous channel activity appeared. Voltage ramp recordings used a 1-s voltage ramp from −150 to +150 mV. Data were filtered at 2.9 kHz (Bessel filter, HEKA amplifier), digitized at 50 kHz, and exported to Clampfit (Molecular Devices) via MatLab (MathWorks). Recordings were analyzed using PatchMaster and Clampfit software. Gaussian curves were fitted to current amplitude histograms, and channel conductances and equilibrium potentials were calculated from linear regressions of the current-voltage relationships.

##### Homology Modeling

The modeling program I-TASSER (40–42) was used to create homology models of CMLV GAAP. The sequence of CMLV GAAP (GenBank^TM^ protein accession number AAG37461.1) was used in structure-based sequence alignments to search for likely structures within the Protein Data Bank. These searches determined that the use of BsYetJ structures as templates gave the best models. The crystal structures of BsYetJ in the closed (Protein Data Bank entry 4PGR) and open (Protein Data Bank entry 4PGS) states were used as templates for the models shown. These models achieved confidence scores (*C*-scores) of −0.2 and −0.07, respectively, that are indicative of correct models, which usually have thresholds of >−1.5 (41).

## RESULTS

### 

#### 

##### vGAAPs and hBI-1 Are Ion Channels

**FIGURE 1.**
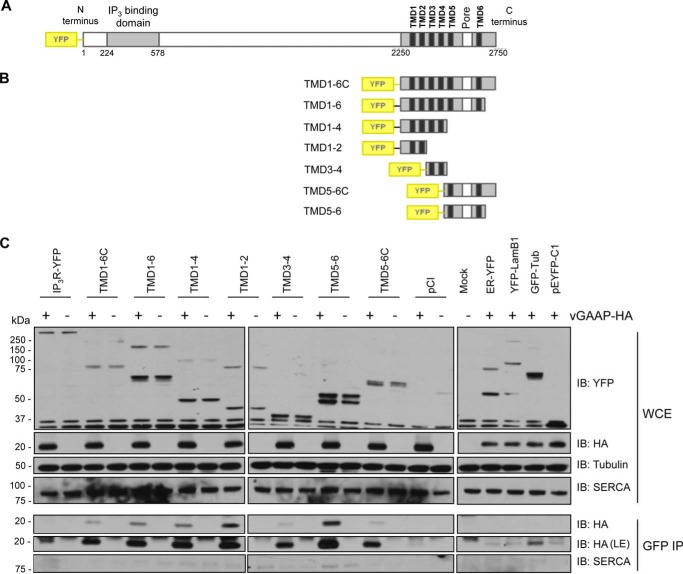
**vGAAP is co-immunoprecipitated with all pairs of TMDs from IP_3_R1.** Shown is co-immunoprecipitation (*IP*) between vGAAP from VACV Evans and full-length and truncated versions of YFP-tagged type 1 IP_3_R. Shown are schematic representations of the full-length (*A*) and truncated forms of YFP-IP_3_R (*B*) used to map the interaction. *C*, COS-7 cells were transfected with plasmids encoding the YFP-IP_3_R proteins, and 18 h later, cells were infected with either v-ΔGAAP (−) or revertant vGAAP-HA (+) VACV and collected after 16 h. Following co-immunoprecipitation with anti-GFP, the immunoprecipitates and the whole cell extracts (*WCE*) were resolved by SDS-PAGE and immunoblotted (*IB*) with anti-YFP, anti-HA, anti-SERCA (as a control for contamination with ER and Golgi membrane proteins), and anti-tubulin (loading control) antibodies. Four additional vectors expressing YFP- or GFP-tagged proteins that localize to different organelles were used as negative controls: ER-YFP (*ER*), YFP-LamB1 (*nucleus*), GFP-Tub (*cytoplasm*), and pEYFP-C1 (*free YFP*). The results shown are typical of three independent experiments. *LE*, longer exposure.

To address the possibility that vGAAP may affect Ca^2+^ homeostasis via an interaction with IP_3_Rs, co-immunoprecipitation analyses were used to test whether the reported interaction of hGAAP with IP_3_Rs also occurs with vGAAP in the context of viral infection and to map the domains of IP_3_Rs involved in the interaction. YFP-tagged type-I IP_3_R (IP_3_R1) and a series of YFP-tagged truncation mutants of IP_3_R1 (33) were expressed in COS-7 cells, followed by infection with either v-ΔGAAP or revertant vGAAP-HA VACV (1) (Fig. 1). YFP- or GFP-tagged proteins that localize to different cellular organelles were included as negative controls. Full-length YFP-IP_3_R1 and fragments containing pairs of its six TMDs all immunoprecipitated vGAAP-HA (Fig. 1*C*), suggesting that these interactions are nonspecific, perhaps due to the hydrophobic nature of these proteins. The inability to detect regions within IP_3_R1 that interacted specifically with vGAAP led us to explore the potential for GAAPs to form ion channels directly.

**FIGURE 2.**
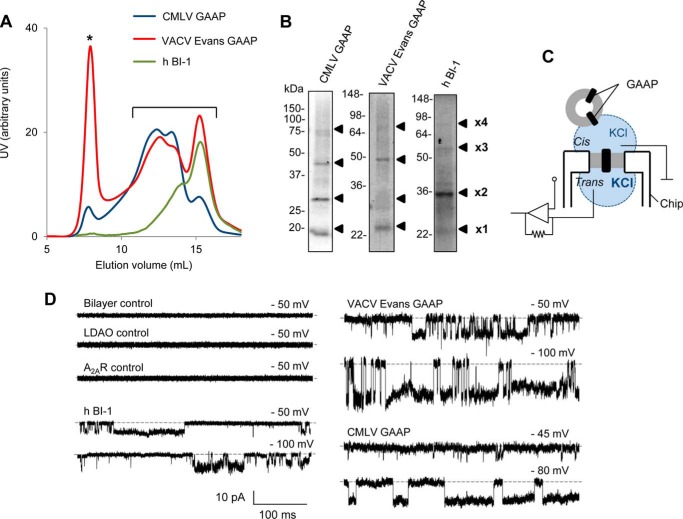
**Purified GAAPs and hBI-1 exhibit ion channel activity in planar lipid bilayers.** *A* and *B*, biochemical analyses of purified CMLV GAAP, VACV Evans GAAP, and hBI-1. *A*, UV absorbance profile of purified vGAAPs and hBI-1 during SEC. *, protein aggregation peak. Fractions corresponding to monomeric and oligomeric populations of vGAAPs and hBI-1 were pooled (*A*, *bracket*) and concentrated, and their contents were analyzed by non-reducing SDS-PAGE and Coomassie staining (*B*). The expected positions of the monomeric (×*1*) and oligomeric proteins (×*2*, ×*3*, and ×*4*) are shown. *C*, bilayer chamber used. A planar lipid bilayer is formed across a micrometer-sized aperture within the chip. GUVs reconstituted with purified protein are added to the *cis* chamber (ground), allowing the incorporation of protein into the bilayer. The KCl concentration is greater in the *trans* relative to the *cis* chamber. *D*, electrophysiological recordings from artificial lipid bilayers reconstituted with purified hBI-1, VACV Evans GAAP, or CMLV GAAP show spontaneous channel openings. Representative current traces were recorded at the indicated holding potentials, which are expressed as the potential on the *cis* side relative to the *trans* side. Downward deflections of the current trace represent positive ions flowing from the *trans* to the *cis* side of the bilayer. The lipid bilayer alone (*n* = 35), after the addition of GUVs reconstituted in the presence of lauryldimethylamine *N*-oxide (*n* = 10), or reconstituted with A_2A_R (*n* = 6) was used as a negative control. *Dotted line*, closed state.

To test whether GAAPs and hBI-1 form ion channels, vGAAPs were purified before electrophysiological analysis in artificial lipid bilayers. vGAAPs from CMLV and the Evans strain of VACV, rather than hGAAP, were chosen for analysis because they were more stable after expression in *S. cerevisiae*. After purification of the proteins expressed in *S. cerevisiae*, SEC (Fig. 2*A*) and non-reducing SDS-PAGE (Fig. 2*B*) revealed the expected presence of three or more oligomeric states of the vGAAPs and hBI-1 (22, 27). The SEC profiles indicated differences in the oligomeric composition of the purified proteins with the smallest (monomers) eluting last (Fig. 2*A*). Calculations of the areas under the curves indicated that CMLV GAAP and VACV GAAP favored oligomeric states (∼87 and ∼64% of total protein, respectively), whereas the monomer was dominant for hBI-1 (∼57% of total protein; Fig. 2*A*). Previous work showed that monomeric vGAAP was anti-apoptotic and reduced the Ca^2+^ content of intracellular stores (27). The functional properties of oligomeric vGAAP are unknown. To optimize opportunities for detecting channel activity, the purified proteins were used for functional reconstitution without separating the monomeric and oligomeric states (Fig. 2*A*, *bracket*).

The experimental conditions used for channel recordings are shown in Fig. 2*C*. Incorporation of purified proteins into GUVs and then into artificial planar bilayers (Fig. 2*C*) gave rise to spontaneous openings of single channels for both vGAAPs and hBI-1 (Fig. 2*D*). These conductances were not observed in untreated lipid bilayers after the addition of GUVs reconstituted in the absence of protein or after the addition of GUVs reconstituted with purified A_2A_R (37), which had been purified from yeast using methods similar to those used to purify vGAAPs and hBI-1 (Fig. 2*D*). These results provide the first direct evidence that vGAAPs form ion channels and that this function is conserved in another member of the TMBIM family, hBI-1.

##### Comparison of the Sequences of GAAPs with Ion Channels Identifies Putative Pore-lining Residues

**FIGURE 3.**
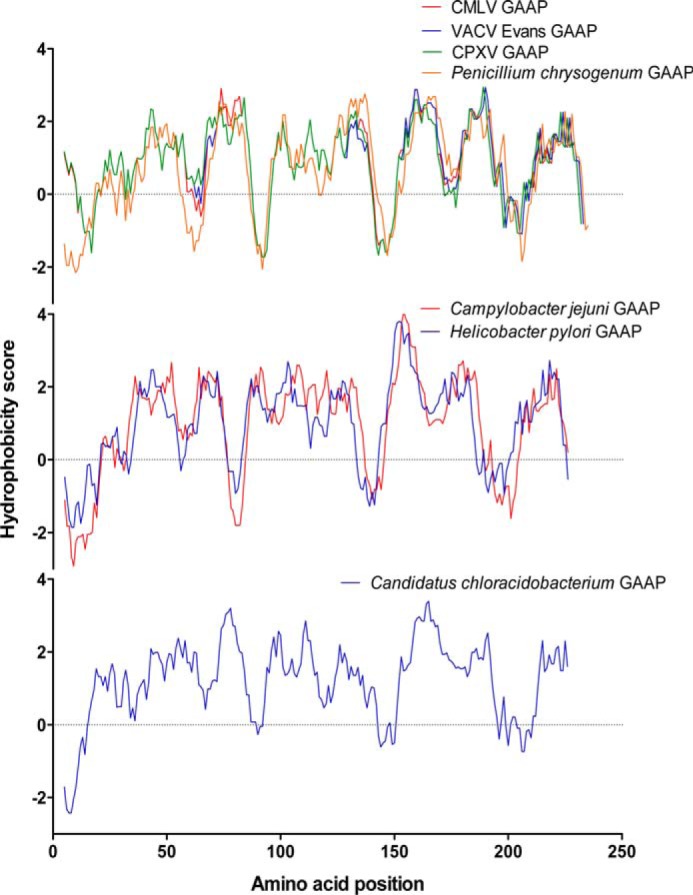
**GAAP hydrophobicity profile is conserved among putative GAAP orthologues from viruses, fungi and bacteria.** Hydrophobicity profiles for VACV GAAP and CMLV GAAP were aligned with those of newly identified putative GAAPs from CPXV, *P. chrysogenum*, *C. jejuni*, *H. pylori*, and *Candidatus C. thermophilum.* Complete amino acid sequences were used apart from the putative GAAP of *P. chrysogenum* origin in which the N-terminal 34-amino acid extension was deleted.

**FIGURE 4.**
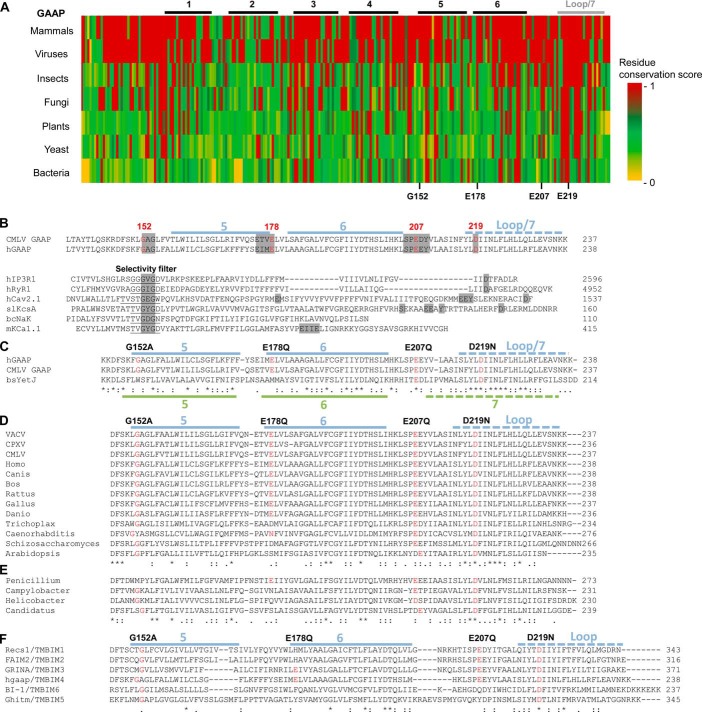
**Comparison of conserved regions within GAAP orthologues and the pore motifs of cation channels reveals a region of greatest sequence conservation toward the C-terminal region of GAAP.** *A*, amino acid sequence alignment of hGAAP against GAAP orthologues from 2–3 representative members from each taxon. The level of conservation for each residue was scored according to Scorecons and represented in a *color gradient*, with *red* and *yellow* indicating identity and no similarity, respectively. Sequences analyzed include *H. sapiens*, *B. taurus*, and *G. gallus* (vertebrates); VACV Evans, CMLV, and CPXV (viruses); *C. biroi* and *T. castaneum* (insects); *P. chrysogenum* and *T. melanosporum* (fungi); *A. thaliana*, *G. aurea*, and *Z. mays* (plants); *S. pombe* and *S. cerevisiae* (yeast); and *C. jejuni*, *H. pylori*, and *Candidatus C. thermophilum* (bacteria). *B–F*, CMLV GAAP and hGAAP from the start of TMD5 to the C terminus were used as queries in BLASTP searches and alignments. The location within CMLV GAAP of TMDs and the proposed re-entrant loop (25) are indicated in *blue*. Residues chosen for mutation in CMLV GAAP are shown in *red. B*, partial alignment of CMLV GAAP and hGAAP with the pore regions of other ion channels. These include the intracellular Ca^2+^ channels, human IP_3_R1 (*hIP_3_R1*), and human ryanodine receptor 1 (*hRyR1*); a human voltage-gated Ca^2+^ channel (*hCa_v_2.1*); the K^+^ channel KcsA from *S. lividans* (*SlKcsA*) and the large conductance Ca^2+^-activated K^+^ channel from *M. musculus* (*mK_Ca_1.1*); and the non-selective cation channel NaK from *B. cereus* (*BcNaK*). The selectivity filters of known ion channels are *underlined*, and similarities between GAAPs and regions of ion channels involved in ion selectivity or conductance are *highlighted* in *gray. C*, partial alignment of the putative pore region of GAAPs with YetJ from *B. subtilis*. The TMDs appearing in the crystal structure of YetJ (28) are mapped over the alignment in *green. D*, alignment of the C-terminal region of representative GAAP orthologues shows the degree of conservation of residues chosen for mutagenesis (*red*). *VACV*, VACV Evans; *Homo*, *H. sapiens*; *Canis*, *C. lupus familiaris*; *Bos*, *B. taurus*; *Rattus*, *R. norvegicus*; *Gallus*, *G. gallus*; *Danio*, *D. rerio*; *Trichoplax*, *T. adhaerens*; *Caenorhabditis*, *C. elegans*; *Schizosaccharomyces*, *S. pombe*; *Arabidopsis*, *A. thaliana. E*, BLASTP analysis using the sequence of VACV Evans GAAP as bait identified putative orthologues of GAAPs in viruses, fungi, and bacteria: cowpox virus (CPXV) strain CPXV_GER2002_MKY_211, *P. chrysogenum* (*Penicillium*) (fungi), *C. jejuni* (*Campylobacter*), *H. pylori* (*Helicobacter*), and *Candidatus C. thermophilum* (*Candidatus*) (bacteria). *F*, amino acid alignment of TMBIM family members shows the high degree of conservation of residues chosen for mutagenesis in CMLV GAAP (*red*). Fully conserved, strongly similar (scoring >0.5), and weakly similar (scoring <0.5) residues are indicated by *asterisks*, *colons*, and *dots*, respectively.

To identify conserved residues within GAAPs that might contribute to formation of an ion channel, we searched for orthologues of GAAP encoded by the genomes of distantly related organisms. BLASTP searches using the sequences of hGAAP and VACV GAAP identified proteins throughout eukaryotes and in CPXV, fungi, and bacteria that share unusually high sequence identity and conserved length for proteins from such distantly related organisms (Table 1). These proteins also have strikingly similar hydrophobicity profiles to vGAAPs (Fig. 3), suggesting that they may be homologues of GAAP. An amino acid conservation plot confirmed the remarkable level of overall conservation within these proteins (Fig. 4*A*). The most conserved residues (*red* in Fig. 4*A*) are within TMD6 and the seventh hydrophobic region. Within the structure of BsYetJ, the seventh hydrophobic region forms a central helix (TMD7) that may line the pore (28), but we have proposed that this hydrophobic region forms a re-entrant loop in vGAAP (25). The subunits of many voltage-gated cation channels also contain 6–7 TMDs and a pore loop near the C terminus (43–45). These observations suggested that residues within the three C-terminal hydrophobic regions of GAAP were likely to contribute to the pore.

**TABLE 1:** **Amino acid identities of known or putative GAAP orthologues** The amino acid (aa) identities of orthologues with VACV Evans GAAP, calculated by the BLASTP server, are indicated.

Known/putative GAAP orthologues	aa identity	aa	Accession no.	Available description
	%			
VACV Evans		237	AAV98625	Golgi anti-apoptotic protein, BAX inhibitor (BI)-1/YccA-like protein family
CPXV	97.5	236	ADZ30397.1	NMDA receptor-like protein
CMLV	95.4	237	AAG37461	6L, GAAP
*H. sapiens*	75	238	AAF14868	S1R protein, z-protein, GAAP
*P. chrysogenum*	34.4	273	XP_002568682.1	Hypothetical protein GAAP-like
*C. chloracidobacterium*	30.5	239	ABV27378.1	Transmembrane BAX inhibitor-1 motif-containing 4
*C. jejuni*	27.7	231	WP_002862428.1	Unknown membrane protein
*H. pylori*	24.8	230	WP_001240234.1	Membrane protein

To identify residues or motifs within GAAP that might be involved in ion selectivity, conductance, or gating, BLASTP searches and sequence alignments with other ion channels were performed using full-length GAAP or individual TMDs. Glycine residues within the pores and selectivity filters of many cation channels facilitate gating (46), whereas both glycine (47, 48) and acidic residues (49, 50) contribute to conduction of ions. A G^152^AG^154^ motif in TMD5 of CMLV GAAP is absolutely conserved among eukaryotic GAAPs (Fig. 4*D*), among members of the TMBIM family (Fig. 4*F*), and in distantly related putative orthologues of GAAP in bacteria (Fig. 4*E*). In coarse sequence alignments, this motif aligned with residues near the selectivity filters of other cation channels (Fig. 4*B*). Likewise, the locations of conserved acidic residues in CMLV GAAP (Glu-178, Glu-207, and Asp-219) roughly aligned with similarly located acidic residues that are known to contribute to ion conduction in other channels (Fig. 4*B*) (49, 50). The conservation of SPE^207^E and D^219^IIN motifs is particularly striking (Fig. 4, *D–F*), appearing in all distantly related proteins in fungi and bacteria (Fig. 4*E*). Four of six TMBIM family members contain Glu-207, whereas all members contain Asp-219 (Fig. 4*F*). Both of these residues are also conserved in BsYetJ (Fig. 4*C*). The residue in BsYetJ (Asp-195) that aligns with Asp-219 of CMLV GAAP is part of a diaspartyl pH sensor that is proposed to keep the channel closed by preventing the movement of TMD2 that is required for channel opening (28). In hBI-1, the equivalent acidic residue (Asp-213) (Fig. 4*F*) is important for the loss of Ca^2+^ from the ER that is evoked by hBI-1 (26).

These analyses suggest that residues Gly-152, Glu-178, Glu-207, and Asp-219 within CMLV GAAP are strong candidates for residues expected to affect its behavior as an ion channel. Therefore, these residues were selected for mutagenesis.

##### Mutation of vGAAP Affects Single-channel Properties

**FIGURE 5.**
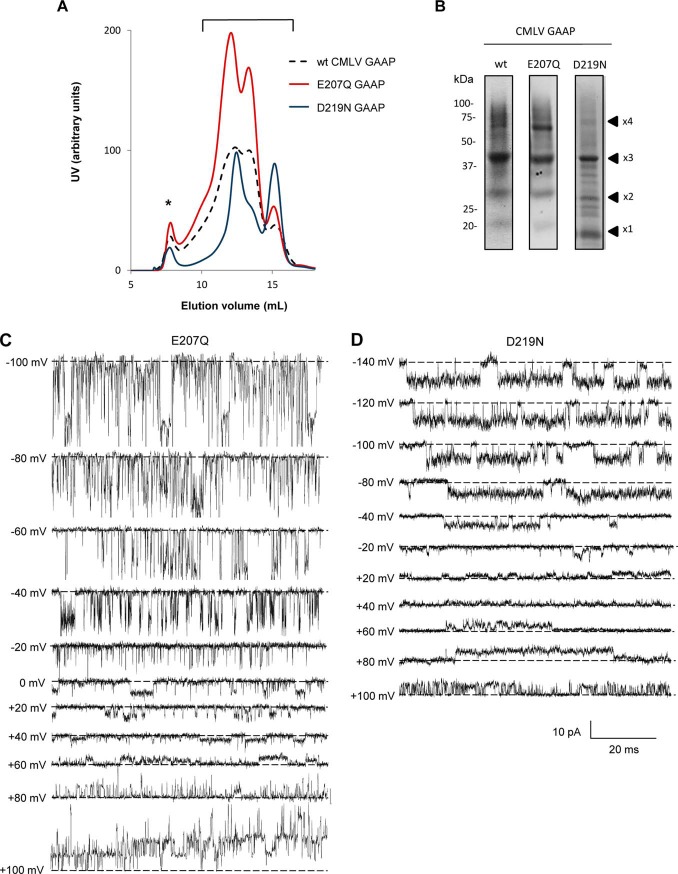
**Mutant vGAAPs form functional channels.** *A*, SEC profile of purified CMLV GAAPs. *, protein aggregation peak. Fractions corresponding to the UV peaks of the various monomeric and oligomeric forms of CMLV GAAP (*bracket*) were pooled and concentrated, and their contents were analyzed (*B*) by non-reducing SDS-PAGE and Imperial staining. The expected positions of the monomeric (×*1*) and oligomeric proteins (×*2*, ×*3*, and ×*4*) are shown. *C* and *D*, representative traces of spontaneous single-channel openings from vGAAPs with the indicated mutations recorded under asymmetric ionic conditions (see “Experimental Procedures”) at the indicated voltages. The closed state is indicated by the *dotted lines*.

To test the effects of the selected point mutations on the electrophysiological properties of GAAP, wild type (WT) CMLV GAAP and the putative pore mutants E207Q and D219N were expressed and purified from yeast. The G152A mutant could not be tested by this method because the protein was unstable after cleavage of the GFP-His_8_ tag used in the purification process. Analysis of the purified mutant proteins by SEC and non-reducing SDS-PAGE confirmed the expected presence of multiple oligomeric states (Fig. 5, *A* and *B*). The relative abundance of the monomer and oligomers differed between the mutants; for WT and E207Q, most protein was oligomeric (∼84 and ∼89%, respectively), whereas for D219N, only ∼66% of the protein formed oligomers (Fig. 5*A*). All of the monomeric and oligomeric states were pooled (Fig. 5*A*, *bracket*) before concentrating the proteins for electrophysiological analyses.

**FIGURE 6.**
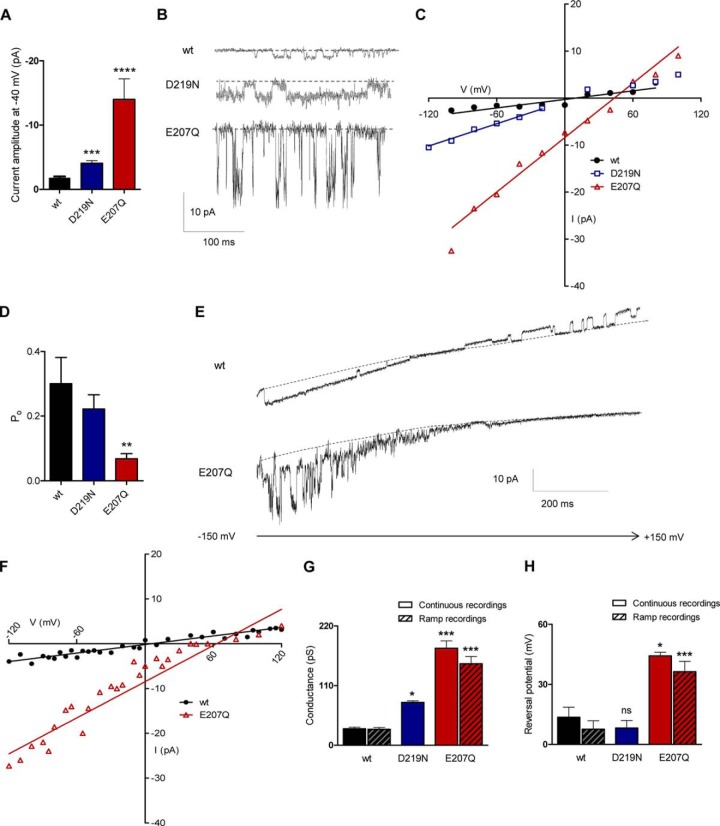
**Mutant vGAAPs have altered single-channel properties.** Spontaneous single-channel currents produced by purified WT, E207Q, and D219N CMLV GAAPs in planar lipid bilayers were measured during stepwise changes in membrane potential (*A–D*) or, for WT and E207Q GAAPs, from repetitive voltage ramps from −150 mV to +150 mV over 1 s (*E* and *F*). *A*, comparison of single-channel currents produced by WT and mutant GAAPs at −40 mV (*n* > 4 independent bilayers). Representative traces are shown in *B*, with the closed state indicated by a *dotted line. C*, current-voltage (*i-V*) relationships for single-channel currents measured at different voltages (*n* = 4–6 independent bilayers). *D*, single-channel open probability (*P*_o_) measured at −40 mV from 4–7 bilayers for WT and mutant CMLV GAAP proteins. Results show means ± S.E. (*error bars*) (**, *p* < 0.01). *E*, representative recordings of WT CMLV GAAP and the E207Q mutant channel activity during voltage ramps. The closed state of the channel is indicated by a *dotted line. F*, the current-voltage relationships of single-channel currents produced by WT CMLV GAAP and mutant E207Q measured from voltage ramp recordings (*n* > 14 independent bilayer recordings). *G*, single-channel conductances calculated from continuous and voltage ramp recordings (*n* = 4–5 and *n* > 14 independent bilayers, respectively). Conductance measurements for the D219N mutant were restricted to negative voltages. *H*, reversal potentials, measured from single-channel currents recorded during stepwise changes in voltage or voltage ramps (*n* = 3–5 and *n* > 8 independent bilayers). *A*, *D*, *G*, and *H*, statistical analyses relative to WT GAAP were made using an unpaired Student's *t* test (*A*) or one-way analysis of variance (*D*, *G*, and *H*) followed by Dunnett's (*D*) or Newman-Keuls multiple comparison tests (*G* and *H*); data shown as means ± S.E. (*, *p* < 0.05; **, *p* < 0.01; ***, *p* < 0.001).

After reconstitution of purified protein into planar lipid bilayers, both the E207Q and D219N mutants of vGAAP generated spontaneous single-channel currents (Fig. 5, *C* and *D*). Relative to WT vGAAP, both mutations increased the amplitude of the single-channel currents recorded at a fixed voltage (Fig. 6, *A* and *B*). Current-voltage (*i-V*) relationships measured from stepwise changes in voltage (Fig. 6*C*) or from voltage ramps (Fig. 6, *E* and *F*) provided consistent results (Fig. 6, *G* and *H*); both mutants caused an increase in the conductance of the single channels (150 ± 13 picosiemens for E207Q, 79 ± 3 picosiemens for D219N, and 29 ± 3 picosiemens for WT vGAAP) (Fig. 6*G*). Under the ionic conditions used (see “Experimental Procedures”), the reversal potential (*V*_rev_) of the single-channel activity produced by WT vGAAP (8 ± 4 mV) (Fig. 6*H*) is consistent with it forming a cation-selective channel. The reversal potential was similar for the D219N mutant (8 ± 4 mV), but it shifted to 36 ± 5 mV for the E207Q mutant (Fig. 6*H*), suggesting an effect of this mutation on ion selectivity. The open probability (*P*_o_) of the E207Q mutant was also altered compared with WT and D219N vGAAPs (Fig. 6*D*). The effects of mutations in vGAAP on single-channel properties establish unequivocally that the observed ion channel activity is attributable to the GAAP protein and not to unknown contaminants. These results demonstrate that mutations of Glu-207 and Asp-219 within vGAAP affect ion selectivity and/or single-channel conductance and suggest that these residues are located within the pore region.

##### Residue Asp-219 of CMLV GAAP Is Required for Protection from Apoptosis

**FIGURE 7.**
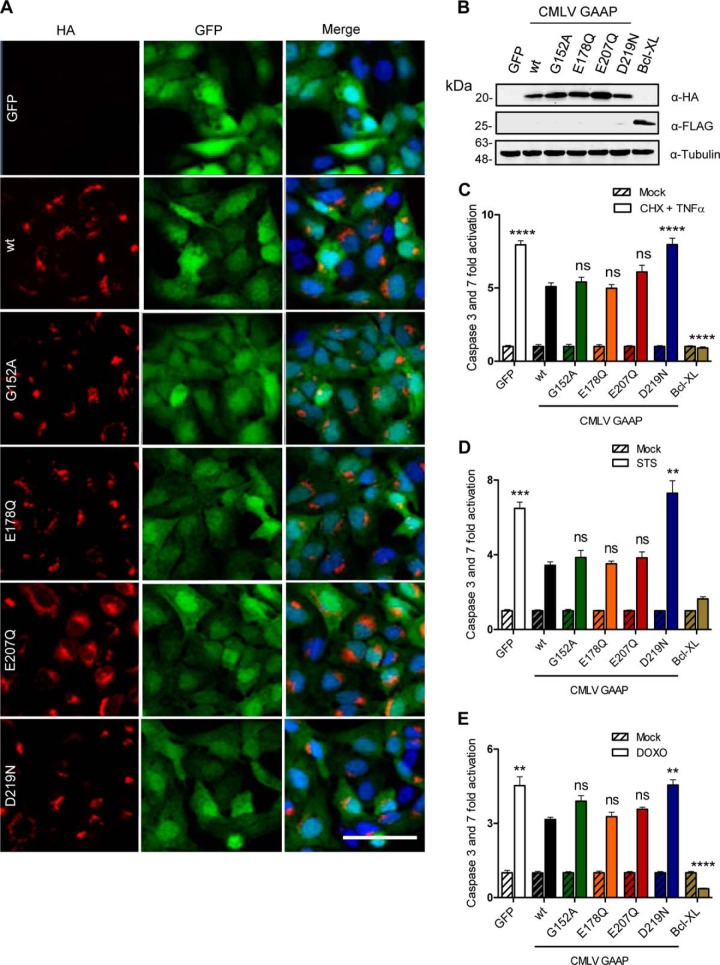
**Residue Asp-219 of CMLV GAAP is required for GAAP-mediated protection from apoptosis.** U2-OS cells were transduced with a bicistronic lentivirus encoding GFP alone or with WT or mutant CMLV GAAPs, each with a C-terminal HA tag. The Bcl-X_L_ lentivirus encoded FLAG-tagged Bcl-X_L_ and GFP. Cells were sorted using fluorescence-activated cell sorting, based on GFP expression. *A*, confocal microscopy of U2-OS lentivirus cell lines fixed and stained with anti-HA antibody. GFP is shown in *green*, and DAPI staining is shown in *blue. Scale bar*, 40 μm. *B*, cell lysates were immunoblotted with anti-HA, anti-FLAG, and anti-tubulin as a loading control. *C–E*, polyclonal U2-OS cell lines were mock-treated or treated with CHX (20 μg/ml) and TNF-α (10 ng/ml) for 16 h (*C*), STS (0.5 μm) for 6 h (*D*), or DOXO (3 μm) for 48 h (*E*), and the activities of caspase-3 and -7 were measured. Results are representative of 3–4 independent experiments. Statistical analyses relative to WT GAAP were made using one-way analysis of variance followed by Bonferroni's multiple comparison test (*C–E*); data are shown as means ± S.E. (*error bars*) (*, *p* < 0.05; **, *p* < 0.01; ***, *p* < 0.001; ****, *p* < 0.0001).

To test the ability of CMLV GAAP and its mutants to inhibit apoptosis, U2-OS polyclonal cell lines were generated using a lentivirus system to deliver a bicistronic vector encoding GFP and CMLV GAAP or its mutants. Lentiviruses encoding GFP alone or in combination with Bcl-X_L_, a potent broad range inhibitor of apoptosis, were included as controls. GFP-expressing cells were selected by fluorescence-activated cell sorting, resulting in almost 100% of the population expressing the gene of interest (Fig. 7*A*). Immunoblotting of cell lysates showed similar levels of expression of WT and mutant CMLV GAAPs (Fig. 7*B*). In addition, the subcellular locations of mutant and WT CMLV GAAPs appeared similar and were consistent with staining patterns of hGAAP and VACV GAAP described previously (1, 25), although E207Q was also visible in the perinuclear region of some cells (Fig. 7*A*).

To assess the ability of CMLV GAAP mutants to inhibit apoptosis, the activities of caspase-3 and caspase-7 were measured after treatment with STS or DOXO to stimulate the intrinsic pathway of apoptosis or with CHX and TNF-α to stimulate the extrinsic pathway. As expected, Bcl-X_L_ and, to a lesser extent, WT CMLV GAAP reduced apoptosis induced by CHX and TNF-α (Fig. 7*C*). Only one of the four mutants of CMLV GAAP (D219N) impaired the ability of GAAP to confer resistance to apoptosis induced by CHX and TNF-α (Fig. 7*C*). The same results were obtained when STS or DOXO was used to trigger apoptosis; only the D219N mutant failed to provide protection (Fig. 7, *D* and *E*, respectively). These results demonstrate that residue Asp-219 of CMLV GAAP is essential for inhibition of apoptosis induced through intrinsic and extrinsic pathways, whereas residues Glu-178, Glu-207, and Gly-152 are not.

##### Residues Glu-178 and Glu-207, but not Asp-219, of CMLV GAAP Are Important for Cell Spreading and Migration

**FIGURE 8.**
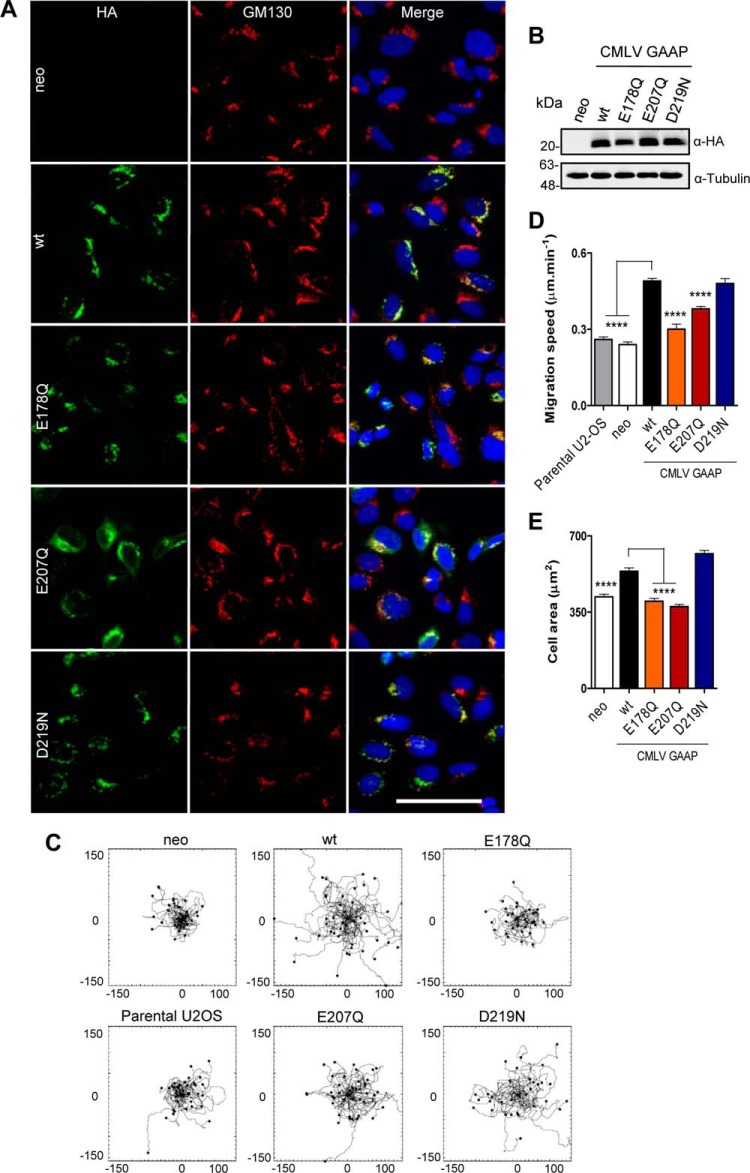
**Residues Glu-178 and Glu-207 are important for the CMLV GAAP-mediated increase in cell migration and spreading.** Polyclonal U2-OS cell lines expressing the empty vector control (*neo*) or WT or mutant CMLV GAAPs with a C-terminal HA tag were selected using neomycin. *A*, cells were imaged by confocal microscopy after fixation and antibody staining of HA and the Golgi marker, GM130. *Scale bar*, 40 μm. *B*, cell lines were immunoblotted using anti-HA and anti-tubulin antibodies. *C* and *D*, cells were allowed to settle for 4 h on fibronectin and then imaged at 5-min intervals for 8 h using wide field phase-contrast microscopy. Tracks of individual cells are shown in *C*, with the scale in micrometers. Cumulative migration speeds from multiple migration tracks (*n* > 40 cells) are shown in *D. E*, cells were left to adhere on fibronectin, and after 30 min, they were fixed, and cell areas in contact with the coverslip were measured for >100 cells in each condition. *D–E*, values are shown as mean ± S.E. (*error bars*); ****, *p* < 0.0001 (one-way analysis of variance followed by Bonferroni's multiple comparison test, relative to WT CMLV GAAP cells).

To test the effects of point mutations in CMLV GAAP on cell adhesion and migration, neomycin-selected polyclonal U2-OS cell lines were generated that overexpressed WT or mutant CMLV GAAP with a C-terminal HA tag (Fig. 8*B*). The speed of random cell migration was assessed, and the areas of cell spreading after seeding were used to measure adhesion and spreading efficiency. Mutation of CMLV GAAPs did not affect their localization at the Golgi apparatus, as shown by co-localization of GAAP with the Golgi marker, GM130 (Fig. 8*A*), although, as seen with the lentivirally transduced cells (Fig. 8*A*), CMLV GAAP E207Q was also visible in the perinuclear region of some cells (Fig. 8*A*).

Cells expressing the empty vector (neo), WT CMLV GAAP, or the mutants E178Q, E207Q, and D219N were seeded onto fibronectin-coated dishes to monitor the migration of individual U2-OS cells. As reported previously for hGAAP (21), cells expressing WT CMLV GAAP migrated faster than neo and parental U2-OS control cells (Fig. 8, *C* and *D*) and also showed enhanced cell spreading (Fig. 8*E*). This demonstrates that increased cell adhesion and migration are features of hGAAP that are conserved in CMLV GAAP. The mutations E178Q and E207Q each abolished the effects of GAAP on cell spreading (Fig. 8*E*) and abolished (E178Q) or substantially attenuated (E207Q) the effects of GAAP on migration speed (Fig. 8*D*). In contrast, GAAP with the D219N mutation had effects similar to those of WT CMLV GAAP on both migration speed and cell spreading (Fig. 8, *C–E*). These results demonstrate that mutations in the putative pore region of CMLV GAAP have different effects; they can affect the anti-apoptotic activity of GAAP (D219N) or its ability to promote cell spreading and migration (E178Q and E207Q).

##### Structural Models of GAAP Define a Putative Pore

**FIGURE 9.**
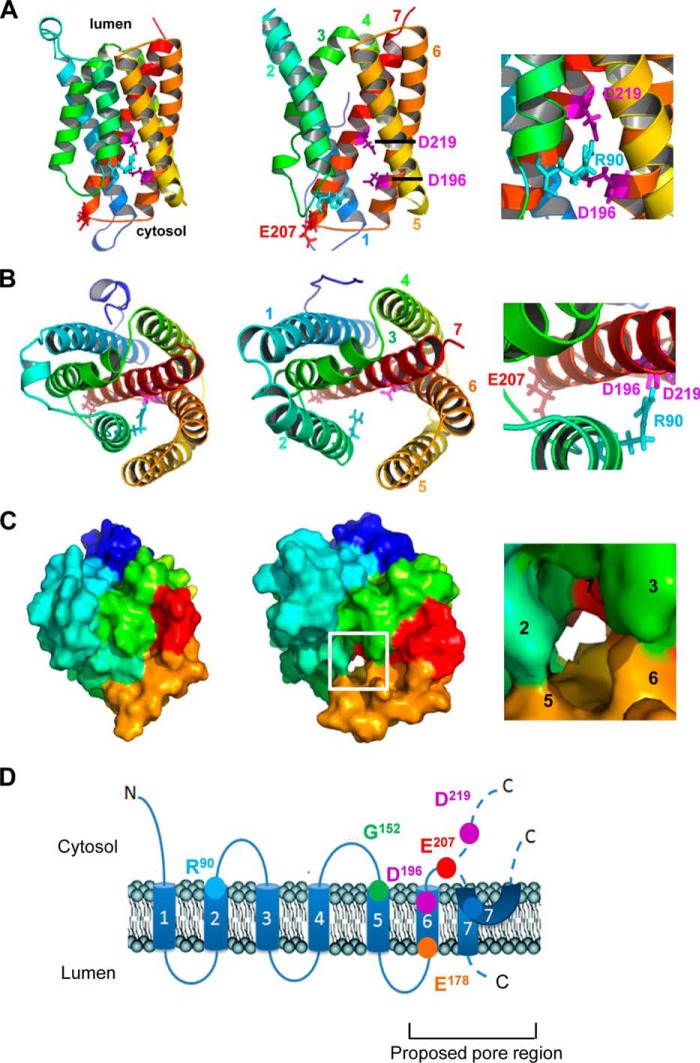
**Structural and topological models of CMLV GAAP.** Homology models of CMLV GAAP based on the crystal structures of BsYetJ were generated using I-TASSER. *A–C*, models of CMLV GAAP in the closed (*left column*) and open (*middle column*) states, viewed from the membrane (*A*) or from the lumen of the Golgi apparatus (*B* and *C*). *A* and *B*, the side chains of residues discussed under “Results” are *colored magenta* (Asp-196 and Asp-219), red (Glu-207), and cyan (Arg-90). Helices are *colored* for clarity: TMD1 (*dark blue*), TMD2 (*light blue*), TMD3 (*green*), TMD4 (*yellow*), TMD5 (*light orange*), TMD6 (*dark orange*), and TMD7 (*red*). The *insets* (*right-hand column*) show *enlarged* regions of the closed state models. Residues discussed under “Results” are labeled. *C*, surface model, with helices *colored* as in *A* and *B*. The *boxed region* of the open state model is enlarged in the *inset* (*right-hand column*), showing the location of a predicted continuous pore across the membrane. TMDs are *numbered* for clarity. *D*, the location of each CMLV GAAP residue is shown *superimposed* over its proposed topological structure (25). The locations of predicted TMD1 to -7 are indicated as shown from a GAAP topological model (25) and from the structure of BsYetJ (28). The biological importance of each residue inferred from data acquired is as follows. Residues Glu-207 and Asp-219 are important for the conductance of the channel and Glu-207 for ion selectivity; thus, both are thought to line the pore of the channel. Asp-219 is essential for protection against apoptosis, whereas Glu-207 is important for cell adhesion spread and migration. Arg-90, Asp-196, and Asp-219 correspond to residues shown to form the channel-closing latch in BsYetJ (28).

Using the crystal structures of BsYetJ in putative closed and open states (28) as templates, we constructed homology models of CMLV GAAP in the closed (Fig. 9, *left column*) and open states (Fig. 9, *middle column*). In the models, Glu-207 and Asp-219 are located at the cytosolic end of TMD7 and near the center of TMD7, respectively (Fig. 9, *A* and *B*). In the closed state model of CMLV GAAP, Asp-196 and Asp-219 are located close to each other within the putative pore and interact with Arg-90 in TMD2 (Fig. 9, *A* and *B*, *left* and *right columns*). This is similar to interactions within the putative closed state structure of BsYetJ (28), where two aspartates in the pore (Asp-171 and Asp-195 of BsYetJ, which align with Asp-196 and Asp-219 of CMLV GAAP, respectively) form salt bridges with each other, and one of these (Asp-171) forms salt bridges with a basic residue in TMD2 (Arg-60 of BsYetJ, which aligns with Arg-90 of CMLV GAAP). In BsYetJ, these interactions stabilize the closed state (28). Protonation of the aspartates (Asp-171 and Asp-195 in BsYetJ) disrupts these interactions, leading to displacement of TMD2 and opening of the channel (28). In the open state model of CMLV GAAP, Asp-196 and Asp-219 are located further from each other and from Arg-90, relative to their locations in the closed state (Fig. 9, *A* and *B*, *middle column*). This is consistent with a disruption of the interactions between these residues during opening of the CMLV GAAP channel. It is perhaps surprising that the mutation D219N failed to increase the *P*_o_ of CMLV GAAP channels (Fig. 6*D*). The mechanisms underlying this effect are unclear. One possibility is that the predicted interactions involving Asp-196, Asp-219, and Arg-90 are already disrupted in WT CMLV GAAP under the experimental conditions used and that additional gating mechanisms exist. Surface models of CMLV GAAP show a predicted continuous pore that traverses the membrane in the open state but not the closed state (Fig. 9*C*). The locations of Glu-207 and Asp-219 at the cytosolic mouth and in the membrane-spanning region of the pore, respectively (Fig. 9, *A* and *B*), show that the acidic side chains of these residues are well placed to affect the passage of cations through the pore. This provides a possible mechanism for the effects of the neutralizing mutations E207Q and D219N on the conductance of single channels.

## DISCUSSION

hGAAP regulates apoptosis, cell adhesion, and Ca^2+^ fluxes, and charged residues near the C terminus of hGAAP have been shown to be important for these effects (1, 20, 21). However, the mechanisms by which GAAP exerts these effects are unresolved. How the related TMBIM protein, hBI-1, exerts its effects on ER Ca^2+^ content and apoptosis is contentious, because BI-1 has been proposed to function as a Ca^2+^/H^+^ antiporter (22, 23) or a Ca^2+^ leak channel (26) or to increase the activity of IP_3_Rs (51, 52). The structure of BsYetJ, a related bacterial protein that shares about 20% amino acid identity with hBI-1 and hGAAP, has been interpreted by speculating that it forms a H^+^-regulated Ca^2+^ channel with a structure unlike that of any known ion channel (28). However, it remains to be confirmed whether BsYetJ forms an ion channel or exchanger (52).

Data presented here demonstrate that hGAAP, vGAAP, and hBI-1 are ion channels and that vGAAP is selective for cations. The spontaneous opening of these channels is consistent with suggestions that the loss of Ca^2+^ from intracellular stores after expression of hGAAP or hBI-1 is due to the activity of a passive leak channel (20, 22). Mutational analyses of amino acids that are conserved within GAAPs and orthologues from eukaryotes and prokaryotes identified residues that affect ion conductance and/or selectivity. Mutation of residues Asp-219 and Glu-207 in CMLV GAAP increased single-channel conductance, whereas E207Q also affected the ionic selectivity. Consistent with our results, mutation of a residue in hBI-1 that is equivalent to Asp-219 (Asp-213 in hBI-1) attenuated the ability of BI-1 to reduce the Ca^2+^ content of the ER (26). The importance of residues Glu-207 and Asp-219 for the function of the pore of GAAP channels, the conservation of these residues among distantly related proteins, and topological data indicating the likely positions of these residues in GAAPs and hBI-1 (25, 28) suggest that the seventh hydrophobic region of GAAP probably lines the pore of the channel (Fig. 9).

The structures of BsYetJ in the pH-regulated open and closed conformations were monomeric (28). A vGAAP mutant that can only form monomers retained its ability to inhibit apoptosis and reduce the Ca^2+^ content of intracellular stores (27). This is consistent with vGAAPs also functioning as monomers, although there is no direct evidence that monomeric GAAP forms a functional channel. Most ion channels are oligomers (50, 53), so the structure of BsYetJ and the activity of monomeric GAAPs is unusual. Nonetheless, both GAAPs and hBI-1 can oligomerize, and this is influenced by pH (22, 27), but the contribution of this oligomerization to ion channel function is unknown. Despite having a topology that is broadly reminiscent of the α-subunits of other ion channels (43, 44), GAAPs lack obvious signature motifs related to selectivity or conductance in other channels (44). This suggests that GAAPs may form channels with novel structures. The large size and 6–7 TMDs of GAAPs from orthopoxviruses make them unique among viral ion channels, and to our knowledge this study constitutes the first report of an ion channel encoded by poxviruses. Viroporins such as M2 channel from influenza virus and the p7 channel from hepatitis C virus are considered to be minimalistic versions of eukaryotic ion channels and typically have only 50–120 residues and no more than 1–3 TMDs (54–60). The lack of structural similarities between BsYetJ and known channels also supports the notion that GAAP channels have a unique mechanism of action (28).

GAAPs and hBI-1 inhibit apoptosis, increase cell spreading and migration speed, and reduce the Ca^2+^ content of intracellular stores. These properties are shared with CMLV GAAP. Whether these effects are independent or the result of a common core function of these proteins has been unclear. Our analyses of mutated residues within the putative pore of vGAAP demonstrate that the effects of GAAP on these activities are separable. Residues Glu-178 and Glu-207 are important for the vGAAP-mediated increase in cell migration and spreading, but not for protection from apoptosis. In contrast, residue Asp-219 is essential for the protective effects of vGAAP against apoptosis but not for enhanced adhesion and migration. Hence, two important biological effects of vGAAP, apoptosis and migration, are differentially susceptible to mutation of two pore-associated residues, Glu-207 and Asp-219. These mutants provide the first tools to study these complex functions in isolation (Table 2 and Fig. 9*D*).

**TABLE 2:**
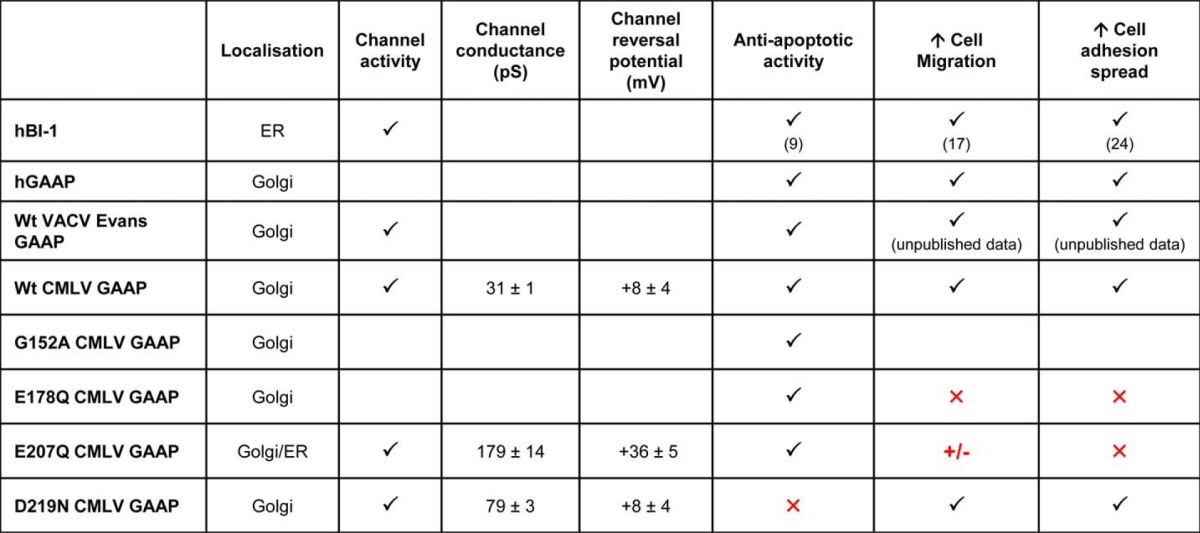
**Summary of data acquired and the biological implications** Shown is a summary of data obtained for hBI-l, GAAPs of different origin, and GAAP mutants. Red annotations indicate residues of biological importance for the different functions of GAAP. Check mark, a WT-like phenotype; +/−, an intermediate phenotype; ×, loss of function.

The localization and characterization of the pore region of GAAP channels described by this study provides insight into their mechanism of action and highlights potential regions that could be targeted by new therapeutics for cancer. Given the ancient origins of GAAP and the remarkable level of topological and amino acid sequence conservation within the TMBIM family, these findings are probably also relevant to other members of the TMBIM family.

## Footnotes

4 The abbreviations used are:

GAAP: Golgi anti-apoptotic protein
hGAAP and vGAAP: human and viral GAAP, respectively
A_2A_R: adenosine A_2A_ receptor
BI-1: Bax inhibitor-1
hBI-1: human BI-1
CHX: cycloheximide
CMLV: camelpox virus
CPXV: cowpox virus
DOXO: doxorubicin
ER: endoplasmic reticulum
GUV: giant unilamellar vesicle
IP_3_R: inositol 1,4,5-trisphosphate receptor
SEC: size exclusion chromatography
SERCA: sarco/endoplasmic reticulum Ca^2+^-ATPase
STS: staurosporine
TMBIM: transmembrane BI-1-motif-containing
TMD: transmembrane domain
VACV: vaccinia virus.
